# Some Observations on Medical Topics in Fiction

**Published:** 1897-03

**Authors:** J. W. Courtney

**Affiliations:** Roxbury


### SOME OBSERVATIONS ON MEDICAL TOPICS IN FICTION.

[Pacific Medical Journal.]

A few days ago while reading a new novel by John Strange Winter, I was very much impressed by a remark made by the married heroine to her husband in reference to her somatic condition—she was pregnant at the time—and I could not help reflecting on the curious medical aspect of life in fiction. For instance, what a charmed—or, if you please, sad—existence the married heroine usually leads! Gifted with a *libido*—if we are to infer anything from the passionate love scenes which take place previous to the marriage—which is only surpassed by the horrible examples in the pages of Krafft-Ebing, she goes merrily along in her butterfly, society life with never, or seldom, an accident in the way of pregnancy to interrupt her pleasure. Her sterility is a thing to marvel at. She may even not be satisfied with her own husband, but her liaisons with other men are just as fruitless. Should the author by any mischance get her pregnant, she usually does not suspect it until very near full term; then it comes upon her like a shock and she communicates her suspicions with much coy hesitancy and in a few well chosen words to her astonished—no, not astonished, that is too mild—to her astounded husband. Such a thing has never entered his head for a moment. When her time has come, the good and skillful old family physician is summoned to my lady's chamber, and in less time than it takes to tell, an heir has come to the vast estates of Slippery-Elmhurst.

The prognostic and therapeutic powers of the unmarried heroine are something to make the keen and experienced specialist shed bitter tears of envy. Her betrothed lover, Count Kickhisownwhiskersoff, is carried almost lifeless from the hunting field, where his spirited steed has thrown him back downward across a fence. The eminent neurologist, Dr. Neuron, is called in consultation, and after a careful and detailed examination pronounces it—fiction M. D.'s always pronounce—a case of fractured spine, and says there is no hope. The poor paraplegic Count goes on from bad to worse under the most skillful hands that money can procure, and *we* can see for him only the usual dismal picture of sphincteric disturbance, bed-sores, cystitis, and the rest. Just before the time when *we* usually get ready to fill out the necessary paper for the undertaker, the heroine takes a hand in the game. Her beautiful, spirituelle face is deadly pale, but there is a gleam of quiet determination in her tear-dimmed eyes. From the side pocket of her elegantly-fitting gown which clings in soft folds to her tall, *svelte* figure, there peeps forth the upper border of a little red-bound volume with a maltese cross in the right-hand corner. "Do not give up hope, dearest," she says in a tone of quiet assurance, "my heart tells me that under the care of your adoring Coccygodynia you will live." The sorrowing relatives silently retire, for now they know that the Count is saved. The next day on making his visit, the doctor is surprised at the marvelous change for the better in his patient, and looks in wonder at the calm, beautiful face of this mere slip of a girl who has installed herself as sole nurse in this most difficult case. How she manages with catheter and bed-pan are totally unimportant and insignificant details—to the author. At the end of a few weeks in such cases—or even less, according to the skill of the individual heroine—the patient is restored to his usual vigorous health, and the virgin heroine joins that wonderfully numerous band of sterile married heroines above mentioned.

The mere simplicity of the therapeutic measures by which these gifted young women bring about such marvelous results is truly astounding. By their sunny smiles alone, children with far-advanced tuberculous joints are restored to blooming health and new joints. Nothing seems to be able to resist them, from advanced phthisis to an impacted gall-stone.

A course of six lectures on "First Insults to the Injured," makes them as skillful as the most practiced surgeon in real life. Ever and anon some sorrowing, but grateful mother of the slums is heard to remark that were it not for the coolness and presence of mind of that angel—one of the above young ladies—in giving Mikey a dose of castor oil when he sucked the ends off the sulphur matches, the dear child would never have had such a speedy and happy death.

With the comparatively few births and the large number of deaths which occur annually in fiction it seems as if the juvenile population must soon be wiped out completely. The life of the child of fiction seems to hang usually by the merest thread. He may look healthy and seem vigorous, but it is merely apparent. Let the society-loving mother neglect such a child for a single evening and its goose is cooked. Some go off the hooks from simple failure to receive the usual good-night kiss. The demise of others is attributed to the careless habit of their otherwise well-meaning fathers of acquiring a jag at the club. A mere cross word spoken in the heat of passion frequently brings about a fatal issue.

When one takes into consideration the extremely delicate health of the ordinary children of fiction and how comparatively small their numbers, it seems strange that their parents can go on killing them off in the grossly careless fashion in which they do. If by any chance, however, the fiction child be vigorous, he is vigorous with a vengeance; his vitality serves the medical reader as an endless source of wonderment. Electric cars may break his legs and fracture his skull, he may fall from dizzy heights, burn almost to a crisp or be buried under any number of tons of coal; he may even be given up by his ordinary medical attendant, but there is always lurking somewhere in the realms of fiction a skilled surgeon to whom such cases are the merest child's play. Truly the advance in fiction surgery in the last few years has been something incredible.

Prognosis, that *pons asinorum* of real life medicine, is just too simple in fiction. There is no heart-rending suspense as to how any particular case is going to turn out; we know from the very start. "A very serious case, my dear madame," says the eminent physician, Sir Willingtew Gullem, "a very serious case, hemorrhage into the medulla, but have no fears; with good care we shall have him about again in a few days."

Verily the penetration of an X-ray is nothing to that of this man's keen mind.

Were I a person of fiction and marked out by my Creator for a few days' illness, there is no disease under heaven I would more willingly elect than a good smart cerebral hemorrhage, or a fractured spine, provided always I could have the attention of a man like Sir Willingtew Gullem.

I will not occupy your time further by multiplying instances of the wonders of medical fiction; the few I have mentioned are enough to make us poor medical men of real life despise our puny efforts to accomplish anything among mere creatures of flesh and blood. Very truly yours,

J. W. COURTNEY, M. D.

*Roxbury, June 10, 1896.*

